# Editorial

**Published:** 1839-02-02

**Authors:** 


### THE MEDICAL EXAMINER.

PHILADELPHIA, FEB. 2, 1839.

We publish to-day the medical evidence delivered in a late criminal trial, with the more important details of the history of the case. We propose to offer some remarks upon the character and value of the necroscopic portion of this evidence. With this view, the attention of our readers is asked to a consideration of the questions, whether, from the testimony of the gentleman who made the autopsy, the facts were established,—1. Of recent delivery. 2. That abortion had been produced—and by violence. 3. That there was adequate cause of death in the uterine appearances.

1. *Were there conclusive signs of recent delivery?* On this point the statement of the witness was, "that, from the condition of the uterus, there could be but one opinion on the subject—that the deceased had been pregnant." From this opinion, founded on a mere examination of the uterus, we must dissent; all or any of the appearances detailed, may have been the result of disease. It is to the appendages of the uterus, particularly to the ovaries, and not to that organ alone, that we are to look for irrefragable evidence, in cases of doubtful pregnancy. If a genuine corpus luteum be found in either ovary, the question may be regarded as settled, though we are still bound to note the condition of all the other organs. In the present instance the ovaries are not even alluded to, nor is mention made of the condition in which the ligaments, fallopian tubes, abdominal integuments, or external organs of generation were found. The appearance of the areola was adduced in proof of the fact of recent pregnancy. The witness stated "that the areola had changed its ordinary colour in the virgin state, to the dark brown, characteristic of the impregnated state." The change in the colour and size of the areola is, however, generally conceded to be a very fallacious sign of pregnancy. In fact, the change in colour is a much less constant and appreciable accompaniment of pregnancy, than the tumid appearance of the areola, and the turgescence of the papilla itself. But the sympathy of the mammæ with the uterus is often either totally absent, or very feebly developed. In genuine blondines, and brunettes, the areola rarely undergoes any appreciable change. It is, moreover, well known that the changes in colour and size, attributed to the areola during pregnancy, are simulated in organic and functional diseases of the uterus; and a late authority, Prof. Hohl, of Bonn, states, that this change occurs in certain females, at every catamenial period. In the present case, it must be borne in mind, that the sign in question was observed after the body had been a week inhumed. At this time, its value,—if it ever possesses any,—must have been materially impaired. No greater importance is to be attached to the lacteal secretion, than to the change in the areola, as an indication of pregnancy. Its occurrence we are disposed to consider as much more frequent, in cases of uterine disease and irritation, than is commonly admitted. None of the autopsic phenomena developed, we are bound to express our conviction, authorized the *judicial* opinion, that a recent delivery had taken place. How the witness was enabled to determine the age of the fœtus, from a cadaveric examination of the female organs, we cannot understand.

2. *Did the autopsic evidence establish the fact that abortion had been produced by violence?* The appearance of the uterus did not warrant the conclusion that there had been premature delivery. The dimensions of the uterus were such as we might expect to find them, at the same period after delivery at term. Admitting, however, the fact of premature delivery to be established by other testimony, the important question presents itself, was there evidence of undue manipulation in the extraction of the fœtus or placenta?

The witness stated that he found "the whole internal surface of the womb and vagina of a bluish-black appearance, as were also the internal genital organs, which, in his opinion, indicates the existence of gangrene." A dark pulpy condition of the lining membrane of the uterus is to be met with, in almost all cases of death after recent delivery, "so that the uterus is thought to be *gangrenous* by those not aware of the circumstance."—(*Burns.*) The appearance of the uterine appendages, too, is a dingy purple. When the internal surface is cleaned, the mucous membrane beneath will be found of natural colour and consistence. This effect followed the immersion of the womb in spirits, in the present case. After the expulsion of the afterbirth, the uterus, in favourable cases, contracts to the size of the fist, and is to be felt a little above the pubes. This is its first action; relaxation now commences, and gradually continues, until the uterus nearly reaches the level of the umbilicus; permanent contraction now commences, and proceeds until the organ regains its unimpregnate size. During this temporary enlargement, its parietes again become soft, and its vessels are filled with blood. It is while the organ is in this condition that the lochial discharge probably takes place, and this ceases when its maximum of contraction occurs. In this state of relaxation, its cavity must contain more or less blood, though not enough to cause alarming hæmorrhage; and hence, in examining women who have perished in child-bed, we must expect to find the internal tunic of the womb coated with a dark, grumous fluid. The appearance of gangrene would be altogether different. The existence of a laceration at the mouth of the uterus, is an item of some importance in the evidence, and would have warranted, perhaps, the witness's statement, that he believed undue manipulation had been exerted, provided the other facts elicited were corroborative. But we feel bound to meet with a direct negation, the assertion, that such an inference was warrantable from the "appearance of the internal coat of the uterus and vagina, and of the internal organs of generation," if they were accurately described by the witness on the trial. We may remark here, that the opinion and experience of the witness, on the risk of retained placenta, are in direct opposition to those of the best obstetrical writers.

3. *Was there adequate cause of death in the ute-*

*rine appearances?* "I saw adequate cause of death from the appearance of the uterus and vagina."—(*Testimony*, page 75.) The narration of the witness is, on this point, neither full nor explicit. There appears, however, to have been peritoneal inflammation, to which we are disposed to ascribe the immediate cause of death. On what did this peritoneal inflammation depend? Was it the result of the laceration at the neck of the uterus, or was it the consequence of intestinal perforation? From the medical evidence, we can only conjecture that it was secondary to the uterine injury, but we have no positive proof that it did not result from perforation of the intestines. The witness did not examine the intestines or rectum, because he found, on a superficial glance at the stomach, that it presented nothing remarkable. One of the counts of the indictment was for the administration of poison, but it was abandoned, from the imperfect autopsic examination. Her own family deposed on the trial, that she had taken medicines to make herself regular, some weeks before her death, and we have her dying declaration that she had taken oil of tansy and pennyroyal, a short time prior to coming to the city. That these took effect, though probably not to an injurious degree, is to be inferred from the deposition of her sisters, that, after using them, she had some bloody discharge from the vagina. How far they were implicated in the final event, we do not pretend to say. The assertion of the witness, that peritoneal inflammation may exist at the time of death, and no trace of it be found upon a post-mortem examination, is incorrect.

The importance of scrupulous minuteness in the performance of judicial autopsies, is forcibly illustrated by the case under examination. From negligence in this particular, many well deserved professional reputations have suffered, both with us and abroad. But the evils which may result from such negligence, are too serious and positive to permit us to pass it by unnoticed. When life and liberty are at stake, every point that may elucidate the subject, *must* be thoroughly investigated. "It is not sufficient to bring proof against the prisoner; it is the duty of the prosecutor to bring the best proof that may be procured; and if any such proof be neglected, the prisoner is entitled to argue that it did not exist; for example, in the present case, that the ovaria were examined, and that no corpus luteum was found, and that all the appearances of pregnancy proved nothing." This is the language of high medico-legal authority,* commenting upon a case parallel to that under notice. Fortunately, in the present instance, there was abundant evidence to elicit the truth, independently of the testimony upon which we have commented. But, in the absence of a dying declaration, of the admission of an accomplice, and of the confession of the prisoner himself, his guilt could not have been established. The evidence educed from the autopsy, proved neither the fact of pregnancy, nor the cause of death.

We shall conclude with a brief abstract of a highly important and interesting trial, which inculcates more strongly than any argument we might adduce, the solemn importance of thorough necroscopic examinations in all cases which may become the subject of judicial investigation, showing, as it does, how totally the ends of justice may be defeated, by the carelessness and ignorance of medical men. Not only are direct hindrances thus opposed to the due administration of the laws, but the physician exposes himself to contempt and derision, and degrades his profession in the public eye.

Charles Angus, Esq., was arraigned at the Lancaster Assizes, England, in 1808, for the murder of Miss Burns, in attempting to procure an abortion by the administration of oil of savine. The prosecution failed to establish the recent delivery of the deceased, and the prisoner was necessarily acquitted. All the signs of recent parturition which were present in Eliza Sowers, were also found in Miss Burns, and the autopsy was conducted by several highly distinguished men. Dr. Carson, the medical witness for the defence, contended that the condition in which the uterus was found might have resulted from the expulsion of hydatids, and as no infallible test of pregnancy was offered by the prosecution, they failed to establish this primary point.

*After the trial* the ovaria were examined, and a genuine corpus luteum was discovered; certificates were then obtained from the most celebrated medical men in London, to the effect that they considered the fact of an advanced stage of pregnancy at the time of death incontestibly proved. This opinion has been confirmed by all recent writers on Forensic Medicine who have noticed the case. Had the gentlemen who were first called discharged their duty, undoubted guilt would not have been permitted to escape, nor

* Edinburgh Medical and Surgical Journal, volume 8, page 229.

their profession have suffered in public estimation.

We propose considering in a subsequent number the interesting subject of medical evidence.

